# High Prevalence of Primary Aldosteronism in Patients with Type 2 Diabetes Mellitus and Hypertension

**DOI:** 10.3390/biomedicines10092308

**Published:** 2022-09-16

**Authors:** Ernestini Tyfoxylou, Nick Voulgaris, Chris Gravvanis, Sophia Vlachou, Athina Markou, Labrini Papanastasiou, Nikolaos Tentolouris, Eva Kassi, Gregory Kaltsas, George P. Chrousos, George P. Piaditis

**Affiliations:** 1 Department of Endocrinology, Diabetes Center, “G. Gennimatas” General Hospital of Athens, 11527 Athens, Greece; 2 Department of Endocrinology, Athens Naval and Veterans Hospital, 11521 Athens, Greece; 3 Department of Endocrinology, 251 Hellenic Air Force & VA General Hospital, 11525 Athens, Greece; 4 Diabetes Center, First Department of Propaedeutic Internal Medicine, Medical School, National and Kapodistrian University of Athens, Laiko General Hospital, 11527 Athens, Greece; 5 Department of Biological Chemistry, Medical School, National and Kapodistrian University of Athens, 15772 Athens, Greece; 6 Endocrine Unit, 1st Department of Propaedeutic Medicine, Laiko Hospital, Medical School, National and Kapodistrian University of Athens, 11527 Athens, Greece; 7 University Research Institute of Maternal and Child Health and Precision Medicine and UNESCO Chair on Adolescent Health Care, National and Kapodistrian University of Athens, 15772 Athens, Greece

**Keywords:** prevalence, primary aldosteronism, type 2 diabetes, hypertension

## Abstract

Primary aldosteronism (PA) is the most common cause of endocrine hypertension. The prevalence of hypertension is higher in patients with diabetes mellitus-2 (DM-2). Following the limited existing data, we prospectively investigated the prevalence of aldosterone excess either as autonomous secretion (PA) or as a hyper-response to stress in hypertensive patients with DM-2 (HDM-2). A total of 137 HDM-2 patients and 61 non-diabetics with essential hypertension who served as controls (EH-C) underwent a combined, overnight diagnostic test, the Dexamethasone–captopril–valsartan test (DCVT) used for the diagnosis of PA and an ultralow dose (0.3 μg) ACTH stimulation test to identify an exaggerated aldosterone response to ACTH stimulation. Twenty-three normotensive individuals served as controls (NC) to define the normal response of aldosterone (ALD) and aldosterone-to-renin ratio (ARR) to the ultralow dose ACTH test. Using post-DCVTALD and ARR from the EH-C, and post-ACTH peak ALD and ARR from the NC, 47 (34.3%) HDM-2 patients were found to have PA, whereas 6 (10.4%) HDM-2 patients without PA (DCVT-negative) exhibited an exaggerated aldosterone response to stress—a prevalence much higher than ever reported. Treatment with mineralocorticoid receptor antagonists (MRAs) induced a significant and permanent reduction of BP in all HDM-2 patients. Early diagnosis and targeted treatment of PA is crucial to prevent any aggravating effect on chronic diabetic complications.

## 1. Introduction

The prevalence of hypertension in patients with type 2 diabetes mellitus (DM-2) is higher than in non-diabetic populations, ranging from 40 to 70% [1]. Hypertension is an additional aggravating factor for the development and progression of diabetes-related micro- and macro-vascular complications, such as diabetic nephropathy and retinopathy and myocardial infarction, congestive heart failure, and stroke [2]. Coexistence of diabetes mellitus with hypertension increases the risk of coronary artery disease (CAD) mortality more than threefold or twofold in younger and older diabetic patients, respectively [3].

Although the role of aldosterone excess in glucose metabolism is not completely delineated, there is substantial evidence supporting such a relation [4]. A moderate increase in basal aldosterone levels by one standard deviation (SD), even within the normal range, has been associated with a 44% higher risk of developing DM-2, whereas in the presence of concomitantly suppressed plasma renin activity levels (PRA ≤ 0.50 ng/L per hour) the risk has been even higher (79%) [3]. Aldosterone excess also impairs the first phase of insulin secretion and may play a crucial role in systemic insulin resistance [5].

Aldosterone excess causes hypertension mainly via two different processes: first, by retaining sodium and water—after binding the cytoplasmic/nuclear mineralocorticoid receptor (MR) in the epithelial cells of the distal convoluted tubules of the kidney—and second, by directly activating the sympathetic nervous system [6,7]. Furthermore, aldosterone excess promotes chronic tissue inflammation, causing fibrosis of vital organs, such as the heart, the blood vessels and the kidneys, potentially leading to cardiac arrhythmias, myocardial infarction, stroke and renal failure. All aldosterone-related complications (renal and cardiac mainly) are more severe and appear earlier than those observed in unselected patients with essential hypertension [8,9].

Although the existing data suggest that aldosterone excess is directly related to abnormalities of the glucose metabolism, data regarding the prevalence of primary aldosteronism (PA) in patients with DM-2 and concurrent hypertension are limited. By contrast, the prevalence of PA has been extensively investigated in unselected hypertensive patients in whom it ranges from 12 to 32%, depending mainly on the severity of hypertension and the diagnostic methodology applied [10,11,12,13,14]. We have shown that 18% of the hypertensive population who suppress aldosterone on standard confirmatory diagnostic tests exhibit aldosterone hypersecretion under conditions of stress, increasing the prevalence of aldosterone excess up to 60% [15]. As the prevalence of hypertension is higher in diabetic than non-diabetic patients [1], a higher prevalence of aldosterone excess in this population would potentially result in aggravation of chronic diabetic complications, which would increase the need for early diagnosis and targeted treatment of aldosterone excess.

**Figure 1:**
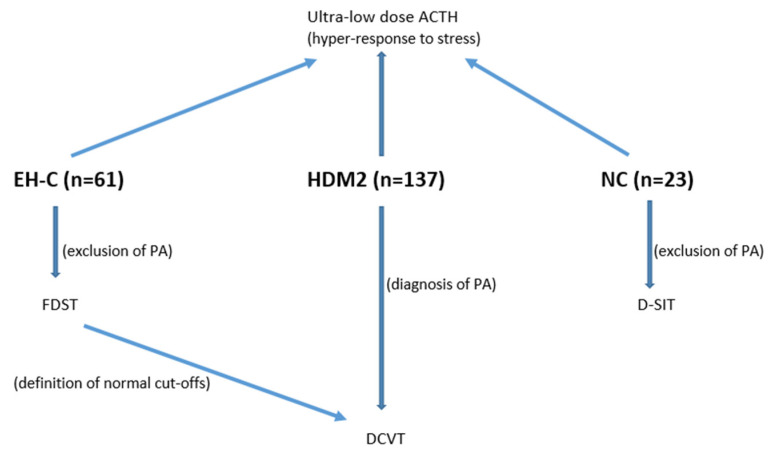
Graphical explanation of the study design. EH-C: non-diabetic patients with essential hypertension; HDM2: hypertensive patients with DM2; NC: normotensive/normoglycemic healthy individuals; FDST: fludrocortisone–dexamethasone saline-loading test; DCVT: dexamethasone–captopril–valsartan test; D-SIT: dexamethasone-saline infusion test.

The aim of this study was to prospectively investigate the prevalence of aldosterone excess, either as a classic autonomous secretion (PA) or as a hyper-response to stress, in patients with DM-2 and concurrent hypertension (HDM-2). Non-diabetics with essential hypertension (EH-C) were also studied as a control group and normotensive/normoglycemic healthy individuals (NC) served as normal controls. For this purpose, we used the following tests: (1) a low-cost overnight diagnostic test of hyperaldosteronism (DCVT), which is easily and safely performed on an outpatient basis in any primary care unit [16], and (2) an ultra-low ACTH aldosterone stimulation test. For the determination of the normal cutoffs, DCVT was performed on EH-C, in whom PA had previously been excluded using the fludrocortisone–dexamethasone saline-loading test (FDST) [12]. The ultra-low ACTH test was performed on all groups in order to detect an aldosterone hyper-response to stress. The upper normal limits of this test were defined from the NC group (Figure 1).

## 2. Materials and Methods

We studied 137 diabetic patients with concurrent hypertension (HDM-2) (78 women, 57%) and 61 non-diabetics (EH-C) with essential hypertension (after exclusion of PA). The group of EH-C patients was age- and sex-matched (37 women, 60.6%) with the HDM-2 patients and was used as a control group. In addition, 23 age- and sex-matched normotensive/normoglycemic healthy individuals (13 women, 61%) with normal aldosterone and cortisol secretory dynamics were used as normal controls (NC) for the definition of normal cutoffs of aldosterone and ARR response to an ultra-low ACTH test. The selected control groups (EH-C and NC) had normal adrenal morphology on computerized tomography (CT). All participants were selected from individuals attending our outpatient endocrine clinic for reasons unrelated to hypertension, mainly thyroid goiter and/or bone mineral density measurements. The adrenal CT scan in controls was performed for reasons not related to hypertension, mainly for nonspecific abdominal pain, renal colic and/or abdominal trauma. All participants were euthyroid. Recruited women were not receiving any hormonal replacement therapy. Exclusion criteria were as follows: pheochromocytoma and other adrenal disease, malignancies, cardiovascular, renal or hepatic disease, pituitary disease, abnormal levels of serum Na, K and N/K ratio in non-hypertensive individuals, and psychological disorders that might cause hypertension. The study protocol was approved by the institutional ethics committee of the General Hospital of Athens “Georgios Gennimatas” (protocol number: 4455/9-2-2017), and informed consent was obtained from all study participants. The reporting of the study conformed to the Strengthening the Reporting of Observational Studies in Epidemiology statement and guidelines.

At initial assessment, the previous medical history was obtained from all participants and recorded and a routine physical examination was performed, including assessment of anthropometric characteristics (weight (kg), height (cm), waist circumference (cm) and body mass index (BMI in kg/m^2^)) by the same physician (E.T.). Arterial hypertension was defined as follows: A. as systolic (SBP) ≥130 mmHg and/or diastolic blood pressure (DBP) ≥80 mmHg, measured at the outpatient clinic for at least three consecutive visits (the mean of the three measurements was calculated), and B. patients who were under antihypertensive treatment already. Any antihypertensive therapy known to affect the renin–aldosterone axis was changed to calcium channel blockers (CCBs) at least 3 weeks before any adrenal hormonal evaluation.

In EH-C controls, PA was excluded by performing the fludrocortisone–dexamethasone saline-loading test (FDST) [12] on an outpatient basis. All participants, including EH-C and HDM-2 patients, underwent the overnight diagnostic dexamethasone–captopril–valsartan test (DCVT) [16]. The two tests in EH-C were performed at least 7–13 days apart from each other. One to four days before testing, depending on the severity of hypertension, the patients did not take their treatment with calcium channel blockers. In addition, in HDM-2 patients without PA (DCVT negative), an ultra-low ACTH test (0.3 μg) was performed aiming at identifying those individuals with an exaggerated aldosterone response (hyper-responders) to ACTH stimulation. To define the normal response of ALD and ARR to the ultra-low ACTH test, 23 normotensive individuals with normal adrenals examined by CT or magnetic resonance imaging (MRI) were recruited and served as controls. Normotensive PA was excluded using the D-SIT (dexamethasone-saline infusion test) diagnostic method [17]. We preferred the use of D-SIT instead of FDST because it was easier to use and, therefore, better acceptable by the NC group.

The ultra-low ACTH test was performed as follows: blood samples were drawn at baseline before the intravenous injection of Synacthen (0.3 micrograms), and then 15 and 30 min later for plasma ALD and renin (REN) measurements and ARR calculation. The HDM-2 hypertensive patients with PA without an adrenal adenoma on CT were all treated with MRAs either as monotherapy or in combination with other anti-hypertensive medications, aiming for SBP < 140 mmHg and DBP < 90mmHg, and for serum K^+^ and renin levels to be within the normal limits. The HDM-2 hypertensive patients with a single adrenal adenoma on CT were offered either medical treatment with MRAs or laparoscopic adrenalectomy.

An adrenal high-resolution CT scan (Philips Brilliance 16 spiral scanner; Best, The Netherlands) was performed consecutively on all subjects studied. Adrenal adenomas were defined as well-circumscribed adrenal lesions with a diameter of more than 10 mm with a non-contrast CT attenuation coefficient of <10 Hounsfield units.

Treatment was offered after the completion of the study in the HDM2 patients with PA, according to the presence or not of an adrenal adenoma and the patients’ preference.

### 2.1. Hormone Assays

Hormones were measured using the following assays: serum cortisol was measured using a radioimmunoassay (RIA) (DIA source Immuno Assays, Nivelle, Belgium). The assay sensitivity for cortisol was 0.08 μg/dl (2.2 nM), and intra- and inter-assay CVs were 6.2% and 8.7% for levels of 0.1 (2.76 nM) and 0.19 μg/dL (5.24 nM), respectively. Plasma REN concentration (renin III generation) was measured by IRMA (CIS biointernational, Gif-Sur-Yvette, France); the sensitivity was 1.8 μU/mL (1.8 mU/L), and intra- and inter-assay CVs were 3.6% and 5% for levels of 6.9 and 7.2 μU/mL (mU/L), respectively. Serum ALD was assessed by RIA (Immunotech assays, Marseille, France); the sensitivity was 0.6 ng/dl (30 pM), and intra- and inter-assay CVs were ≤95% and ≤99%, respectively. Serum ACTH was measured by immunoradiometric assay (IRMA) (CIS biointernational); the sensitivity was 2 pg/mL (0.44 pM), and intra- and inter-assay CVs were 6.1% and 5.3% for levels of 22 pg/mL (4.84 pM) and 40 pg/mL (8.8 pM), respectively.

### 2.2. Statistical Analyses

Statistical analyses were performed using the SPSS software package (SPSS Inc., version 23, Chicago, IL, USA). Continuous variables are presented as mean ± SD. Normality of the data was tested according to the Kolmogorov–Smirnov test. Student’s t-test and the Mann–Whitney U-test were used to analyze continuous variables with and without normal distribution, respectively. One-way ANOVA was performed followed by post-hoc least significant difference (LSD) calculation, which was used for the post-hoc statistical multiple comparison of hormone values in the three different groups (HDM-2, EH-C and NC). The chi-squared test was used for comparison of categorical variables. Pearson and Spearman correlation coefficients were used to evaluate correlations between continuous variables. Univariate and multivariate linear regression models were performed to search for variables associated with eGFR (age, duration of hypertension, of DM, or ALD-post-DCVT). In all cases, a two-tailed *p*-value < 0.05 was considered statistically significant. 

## 3. Results

### 3.1. Patient Characteristics

Participants’ anthropometric characteristics and biochemical profile are shown in Table 1. There were no significant differences regarding age, SBP, DBP, serum K^+^ and Na^+^ levels, and urinary Na^+^ excretion between HDM-2 patients and EH-Cs, whereas urinary K^+^ and K^+^/Na^+^ ratio were significantly higher in HDM-2 patients than in EH-Cs. As expected, BMI, HbA1c and blood glucose (BG) measurements were also significantly higher in HDM-2 patients than in EH-Cs (*p* = 0.0004, *p* < 0.0001 and *p* < 0.0001, respectively).

**Figure 2:**
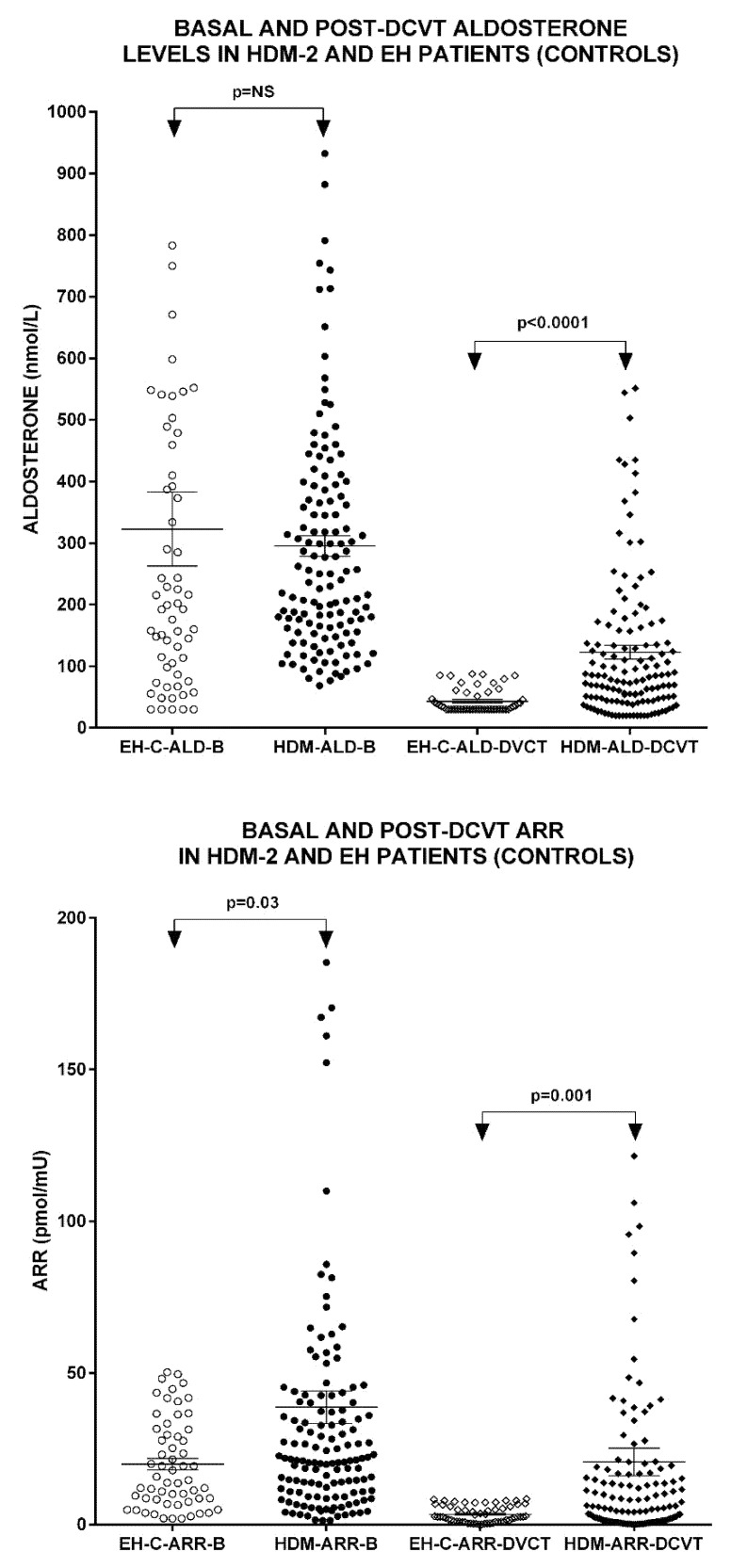
Basal and post-DCVT aldosterone levels (upper panel) and ARR (lower panel) in DM-2 and EH patients. EH: essential hypertensive patients. HDM: hypertensive diabetes mellitus patients.

Basal and post-DCVT hormone levels in HDM-2 and EH-C patients are shown in Table 2. No significant differences were observed regarding basal ACTH, F, ALD, REN and post-DCVT ACTH and REN levels between the two groups, whereas significantly higher basal ARR and POST-DCVTALD levels and ARR ratio were found in the HDM-2 patients compared to EH-Cs (*p* = 0.02, *p* < 0.0001 and *p* = 0.001, respectively) (Figure 2).

### 3.2. Normal Cutoffs for DCVT

In EH-Cs, PA was excluded by performing in all of them the PA diagnostic test FDST. The PA diagnosis is made simultaneously considering the upper-normal cutoffs of post-FDST ALD levels (85 pmol/L) and ARR (26 pmol/mU), as defined in a previous study [12]. In this study, the PA diagnosis in EH-C patients, who served as controls, was excluded, as none of them had both post-FDST ALD and ARR values above the upper normal cutoffs. Using the EH-C patients as controls, the DCVT was also performed in all of them and the 97.5 percentiles were applied to calculate the upper normal cutoffs for basal ARR and POST-DCVT ALD levels and ARR; basal ARR: 64 pmol/mU, post-DCVT ALD: 87 pmol/L and post-DCVT ARR: 9 pmol/mU.

### 3.3. PA Prevalence

To calculate the prevalence of PA in HDM-2 patients, we employed the combination of post-DCVT ALD levels and ARR simultaneously. Based on these cutoffs, 47/137 (34.3%) HDM-2 patients were found to have PA, whereas only 23 (16.7%) had a raised basal ARR ratio and, therefore, were considered as patients with suspected PA. From these 23 HDM-2 patients with suspected PA, only 17 (12.4%) had the PA diagnosis confirmed by DCVT (Table 3).

### 3.4. Hyper-Response to ACTH Stimulation

The normal cutoffs for aldosterone response to ultra-low ACTH administration were obtained from the NC group of participants based on the combination of post-ACTH peak ALD and ARR levels simultaneously. On the basis of the 97.5-percentile values from the control population, the normal cutoff post-ACTH ALD and post-ACTH ARR levels were defined as 900 pmol/L and 79 pmol/mU, respectively. Τhe prevalence of aldosterone hyper-response in DM-2 patients without PA (DCVT-negative) was 6 out of 58 (10.30%) patients who accepted to have the ACTH-test.

### 3.5. Correlations

A considerable number of significant correlations were observed. Positive correlations were observed between basal or post-DCVT ALD levels and the duration (years) of hypertension (r = 0.363, *p* = 0.01 and r = 0.370, *p* = 0.01, respectively), as well as the ALD response (30 min) to ACTH stimulation (r = 0.747, *p* < 0.0001 and r = 0.387, *p* = 0.01, respectively). Basal and post-DCVT ARR were also significantly correlated with the ARR response to ACTH stimulation (r = 0.970, *p* < 0.0001 and r = 0.930, *p* < 0.0001, respectively) in the 58 HDM-2 patients who accepted to have the ACTH test. 

**Figure 3:**
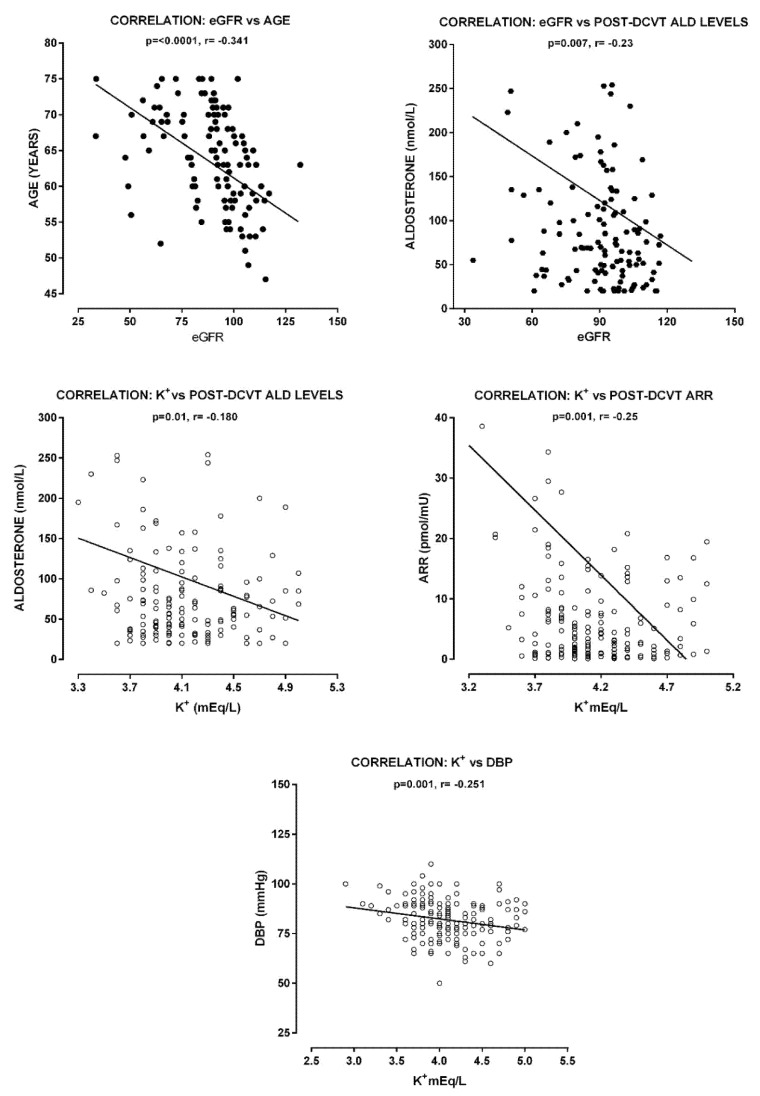
Correlations of eGFR with age and post-DCVT aldosterone levels (**upper panel**). Correlations of serum K^+^ levels with DBP, post-DCVT aldosterone levels and post-DCVT-ARR (**lower panels**).

Negative correlations were observed between serum K^+^ and the post-DCVT ALD levels (r = −0.210, *p* < 0.01), post-DCVT ARR (r = −0.190, *p* = 0.03) and DBP (*p* = 0.001, r = −0.251) in the entire group of patients (Figure 2). Interestingly, the eGFR was significantly correlated with patients’ age (r = −0.510, *p* < 0.01), duration of arterial hypertension (r= −0.233, *p*= 0.006) and duration of DM (r = −0.211, *p* = 0.015), as well as post-DCVT ALD levels (r= −0.207, p= 0.015). In the univariate linear regression model, age (b = −0.409, *p* < 0.01), years of hypertension (b = −0.209, *p=* −0.014), years of DM (b = −0.218, *p* = 0.01) and post-DCVT ALD levels (b = −0.229, *p* = 0.007) were independent predictors of eGFR, whereas in the multivariate model, only age (b = −0.383, *p* < 0.001) and post-DCVT ALD levels (b = −0.185, *p* = 0.026) still predicted eGFR status (Figure 3).

### 3.6. Treatment

**Figure 4:**
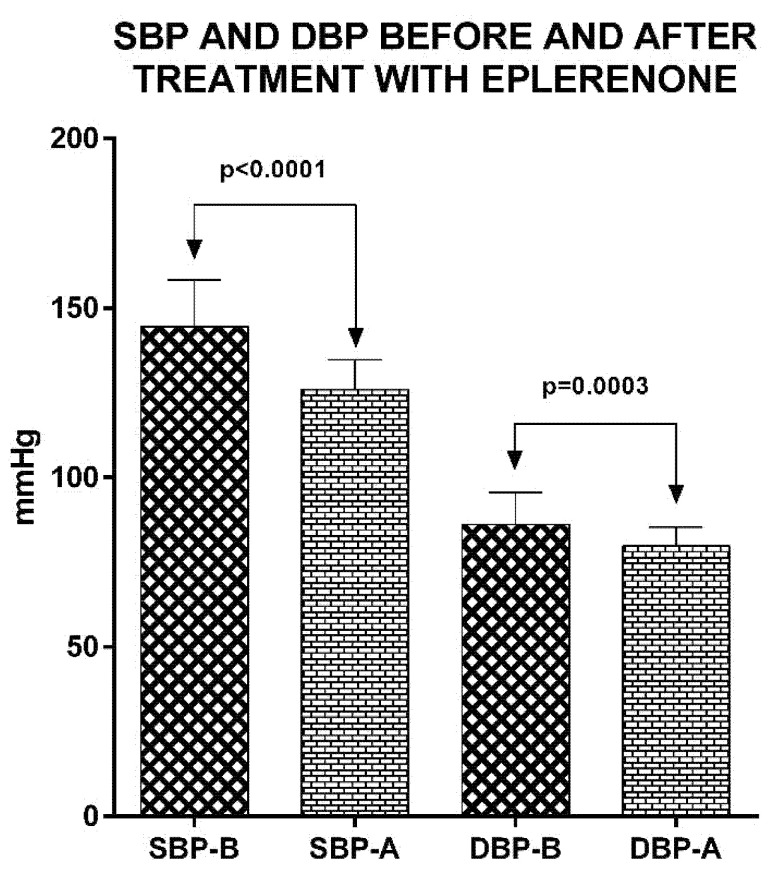
Systolic blood pressure (SBP) and diastolic blood pressure (DBP) before and after treatment with eplerenone either alone or in combination with other anti-hypertensive drugs.

Eight (17%) of the HDM-2 patients with PA had a single adrenal adenoma on CT or MRI imaging with a size ranging from 1 to 4.5 cm. In the remaining 39 (83%) the adrenal imaging was normal and they were considered having adrenal hyperplasia. All 47 HDM-2 patients were treated with MRAs alone or in combination with other anti-hypertensive medications, as none of the eight patients with a single adrenal adenoma elected to have laparoscopic adrenalectomy. Among the DM-2 patients, 16 (34%) were treated with eplerenone alone, 18 (18.3%) with eplerenone and a calcium channel blocker (CCB), 4 (8.5%) with eplerenone and a β-adrenergic blocker, 6 (12.8%) with eplerenone, a CCB and a β-adrenergic blocker and 3 (6.4%) with a β-adrenergic blocker alone. All HDM-2 patients with PA were followed up to 11 ± 1.7 (min: 9, max: 14) months after the treatment with MRAs was initiated and the final clinical and biochemical assessment was performed in the last visit (Table 4). Treatment induced a significant and stable reduction of BP in all HDM-2 patients, which was restored to normal (SBP < 140 mmHg, DBP < 90 mmHg) in 45 (95.7%) (Figure 4). Serum K^+^ and renin levels were within normal limits in all of them. Two of the HDM-2 patients failed to have their BP and renin levels restored to normal and a further increase in eplerenone dose was applied.

## 4. Discussion

The results of this study show a prevalence of PA in patients with HDM-2 that is much higher (34.3%) than ever reported, which has ranged from 0.0 to 22.4% [18,19,20,21,22,23,24]. In addition, an exaggerated aldosterone response to stress through ACTH administration was observed in another 10.3% of the tested HDM-2 non-PA patients. Overall, about 46% of HDM-2 patients exhibited an increased aldosterone secretion. An exaggerated response to ACTH was reported in a previous study of ours in unselected non-diabetic patients with essential hypertension [25]. Similar results were recently reported by Brown et al., using as a diagnostic tool of PA the 24 h urinary aldosterone excretion rate, following an oral sodium loading test [14].

Apparently, the large differences regarding the prevalence of PA between the published studies are mainly based on the different diagnostic methods used and the different populations recruited with a PA diagnosis. In the first published study in which only the basal plasma aldosterone concentration/plasma renin activity (PAC/PRA) ratio (>70) was used for the diagnosis of PA, no PA cases were detected among 61 HDM-2 patients [18]. In other published studies, the PA prevalence in HDM-2 ranged between 4.1% and 22.4% [19,20,21,22,23,24]. In these studies, the diagnosis of PA was based on the standard methodology initially using as a screening test the basal PAC/PRA ratio and as confirmatory test mainly the 2 lt saline infusion. This choice significantly reduces the sensitivity of these diagnostic tests because they aim to suppress only the renin-angiotensin II-system, leaving the secretion of ACTH unaffected [17].

By contrast, in our study we used a highly sensitive and specific diagnostic test, the DCVT, which is based on the pharmaceutical blockade of both main physiological hormonal stimuli of aldosterone, i.e., renin-angiotensin II and ACTH, and has been validated with an oral saline loading test, the modified FDST [16]. In addition, DCVT has already been used in previous studies in non-diabetic hypertensive populations with similar results [13]. The reliability of DCVT is supported by the observation made in this as well as in previous studies, that both post-DCVT aldosterone levels and ARR were negatively correlated with serum K^+^ levels, which are highly dependent on aldosterone action. It is further supported by the observation that the BP, serum K^+^ and renin levels in HDM-2 patient with PA diagnosed by DCVT were restored to normal after treatment with eplerenone either alone or in combination with other anti-hypertensive medications.

An interesting finding of our study was the significant rise in both aldosterone levels and ARR following the ultra-low ACTH test, which were highly correlated with the post-DCVT aldosterone levels and ARR. This finding is in complete agreement with the well-known fact that ACTH is a significant stimulating factor of aldosterone secretion and supports our decision to incorporate into the DCVT—apart from the ACE-I and AT1-blockade—dexamethasone, in order to both block RAAS activity and simultaneously suppress the circulating ACTH levels. This modification reduces the normal cutoffs and significantly improves the sensitivity of the DCVT leading to detection of milder PA forms and leading, therefore, to a greater prevalence of PA in hypertensive populations [12,13,25].

The observed high aldosterone activity in this study is, perhaps, a very important observation, as according to published data, such high aldosterone activity is expected to aggravate peripheral tissue damage, primarily fibrosis, by acting in combination with the metabolic disorders related to DM-2. It is well-known that aldosterone exerts its harmful effect either by raising the blood pressure or by acting directly on various peripheral tissues [6]. It is also well-known that the prevalence of hypertension in patients with DM-2 is much higher than in patients without diabetes [1,26,27]. The age-adjusted prevalence of hypertension in US adults with diabetes is two times higher (57.3%) than in those without diabetes (28.6%). In addition, the coexistence of hypertension in patients with DM-2 greatly enhances the likelihood of their developing cardiovascular (CVD) and chronic kidney disease (CKD) [26,28].

Epidemiological observations have demonstrated that there is a progressive increase in the risk of macrovascular and microvascular events with increasing levels of systolic blood pressure, starting as low as 115 mmHg, considering hypertension as an independent risk factor for the development of DM-2 complications [26,29]. However, despite the administration of the appropriate treatment recommended by the international forums, the blood pressure is poorly controlled in a significant proportion (50%) of HDM-2 patients and the macrovascular and microvascular events remain to a great extent an unresolved problem [30].

The cause(s) for the high rate of failure to control blood pressure in hypertensive patients with DM-2 is not clear. Although a number of different factors have been implicated, it is generally accepted that the chronic activation of the renin–angiotensin–aldosterone system (RAAS) due to Ang II stimulation is the most important cause of hypertension in DM-2 [4]. However, this study showed that about 45% of the hypertensive patients with HDM-2 have increased aldosterone secretion, mainly as autonomous production, which should be a reasonable explanation for the current high rate of failure to control blood pressure using the recommended treatment with ACE-I or AT1-B inhibitors either alone or in combination with other agents [30]. This is in complete agreement with the way of action of these therapeutic agents, as both are usually effective to block RAAS only in case it is functionally activated by Ang-II, whereas they remain ineffective in the presence of autonomous aldosterone production (AAP), which is independent of RAS activity. 

There is increasing evidence supporting the concept that the AAP may also be an important cause of hypertension and peripheral tissue damage in DM-2. Data from many studies support that mineralocorticoid receptor overactivation plays a crucial role in the development and progression of CVD, CKD and diabetes, through inflammation and fibrosis, which are improved with MRA administration [31]. Kidney biopsies from DM-2 patients have shown that only about a third of them have renal histology typical of diabetic nephropathy. Another third has a near-normal renal structure and a further third shows non-specific interstitial fibrosis and arteriolar hyalinosis. These histological changes resemble those induced by hypertension and direct aldosterone action, which are also seen in the cardiovascular system of patients with primary aldosteronism [32]. Furthermore, Bakris et al. published recently that the addition of the non-steroidal mineralocorticoid receptor antagonist finerenone in HDM-2 patients with CKD already treated with maximum doses of either ACE-I or AT-1B induced a further decrease in risks of CKD progression and cardiovascular events [33]. In addition, they found that aldosterone levels were inversely proportional to the GFR and were also associated with inflammation. This is in complete agreement with the results of this study, which showed a significant positive correlation of calculated eGFR in HDM-2 patients with post-DCVT aldosterone levels, as well as with the high prevalence of PA in HDM-2 hypertensive patients in this study. Moreover, the targeted treatment with eplerenone, either alone or in combination with other anti-hypertensive drugs, restored to normal the BP, serum K^+^ and renin levels.

## 5. Conclusions

In this study we found that the prevalence of aldosterone excess, either as classic autonomous aldosterone secretion (34.3%) or as a hyper-response to stress (10.3%) in patients with HDM-2, is higher than previously reported. This finding suggests that aldosterone excess, acting synergistically with metabolic changes related to diabetes mellitus, may aggravate the chronic diabetic complications, emphasizing the need for early diagnosis and targeted treatment. It should be noted that the diagnosis of hyperaldosteronism was made using a rapid, safe, easy and cost-effective test, the DCVT, which can be readily performed in any primary care unit.

## Figures and Tables

**Table 1:** Anthropometric characteristics and biochemical parameters of all hypertensive patients with DM-2 (H-DM-2), patients with essential hypertension (EH-C), who were used as controls for DCVT, and normotensive individuals used as controls in the ultralow ACTH test (NC).

Parameter	HDM-2 (Mean ± SD)	EH-C (Mean ± SD)	NC (Mean ± SD)	*p*1 HDM-2 vs. EH-C	*p*2 HDM-2 vs. NC	*p*3 EH-C vs. NC
N	137	61	23			
Age (years)	63.01 ± 8.45	61.61 ± 7.74	61.70 ± 7.20	NS	NS	NS
SBP (mmHg)	142.10 ± 11.52	136.10 ± 21.58	116.40 ± 8.00	NS	0.0001 **	0.0001 **
DBP (mmHg)	82.76 ± 10.82	80.90 ± 11.48	76.50 ± 5.50	NS	0.0001 **	0.0001 **
BMI (kg/m2)	32.22 ± 5.80	29.25 ± 4.46	28.90 ± 6.50	0.001 *	0.01 *	NS
HBA1c (%)	6.54 ± 0.77	5.58 ± 0.48	5.60 ± 0.40	0.0001 **	0.0001 **	NS
BG (mmol/L)	6.75 ± 1.84	4.95 ± 0.62	5.03 ± 0.42	0.0001 **	0.0001 **	NS
Na^+^ (mEq/L)	139.40 ± 1.94	139.70 ± 1.60	141.10 ± 2.60	NS	0.01	NS
K^+^ (mEq/L)	4.08 ± 0.42	4.05 ± 0.30	4.20 ± 0.30	NS	NS	NS
UrNa^+^ (mEq/24 h)	146.20 ± 62.30	139.3 ± 56.73	121.00 ± 26.00	NS	NS	NS
UrK^+^ (mEq/24 h)	65.55 ± 22.90	56.08 ± 20.25	49.00 ± 17.30	0.01 *	0.001 *	NS
UrK^+^/Na^+^	0.50 ± 0.20	0.42 ± 0.14	0.40 ± 1.20	0.02 *	0.01 *	NS

N: number of patients; SBP: systolic blood pressure; DBP: diastolic blood pressure; BMI: body mass index; UrNa^+^, UrK^+^, UrK^+^/Na^+^, 24-h urinary Na^+^, K^+^ and K^+^/Na^+^ ratio, * significant, ** highly significant “*p*” value.

**Table 2:** Hormonal levels in basal and post-DCVT among all hypertensive patients with DM-2 (HDM-2) and patients with essential hypertension (EH-C), who served as controls.

Hormonal Values	EH-C (Mean ± SD)	HDM-2 (Mean ± SD)	*p*
N	61	137	
BASAL ALD (pmol/L)	322.50 ± 464.20	295.10 ± 196.10	0.1
BASAL REN (mIU/L)	16.47 ± 13.67	14.83 ± 13.18	0.1
BASAL ARR (pmol/mIU)	19.92 ± 14.67	38.72 ± 63.20	0.02 *
BASAL F (nmol/L)	377.10 ± 141.10	370.60 ± 133.70	0.9
BASAL ACTH (pmol/L)	4.94 ± 3.44	4.49 ± 2.62	0.8
Post-DCVTALD (pmol/L)	43.21 ± 19.33	122.70 ± 130.70	<0.0001 **
Post-DCVT REN (mlU/L)	58.80 ± 103.20	37.84 ± 59.89	0.5
Post-DCVT ARR (pmol/mIU)	3.40 ± 2.73	20.69 ± 52.55	0.001 *
Post-DCVT F (nmol/L)	47.41 ± 0.44	56.60 ± 27.48	0.04 *
Post-DCVT ACTH (pmol/L)	1.32 ± 1.98	1.39 ± 0.28	0.2

N: number of patients; ALD: aldosterone; REN: renin; ARR: aldosterone/renin ratio; F: cortisol; ACTH: adrenocorticotropic hormone; post-DCVTALD and post-DCVTARR: aldosterone and aldosterone/renin ratio post-DCVT, * significant, ** highly significant “*p*” value.

**Table 3:** Normal cutoffs, performance characteristic of the tests, and PA prevalence in DM-2 patients.

Hormonal Values	EH Upper Normal Cutoffs	PA-DM-2 with >Upper Normal Cutoff	EH-DM-2 with <Upper Normal Cutoff	Sensitivity %	Specificity %	Prevalence % of PA in DM-2 Patients
BASAL ARR pmol/mU	64	17/47	83/90	36.20	92.20	12.40 (17/137)
POST-DCVT ALD pmol/L	87	47/47	76/90	100	84.50	34.30 (47/137)
POST-DCVT ARR pmol/mU	9	47/47	80/90	100	88.90	34.30 (47/137)
POST-DCVT ALD pmol/L and ARR pmol/mU	87/9	47/47	90/90	100	100	34.30 (47/137)

ALD: aldosterone; ARR: aldosterone/renin ratio; DCVT: dexamethasone–captopril–valsartan test.

**Table 4:** Clinical and biochemical assessment of HDM-2 patients with PA before and after treatment with eplerenone.

	Before Treatment	After Treatment	
Follow-Up Parameters	Mean ± SD	Mean ± SD	*p*
SBP (mmHg)	144.6 ± 13.9	126.0 ± 8.7	<0.0001 **
DBP (mmHg)	86.2 ± 9.4	79.7 ± 5.7	<0.0001 **
Serum K^+^(mEq/L)	3.9 ± 0.4	4.4 ± 0.4	<0.0001 **
Renin (mIU/L)	8.0 ± 5.9	18.4 ± 13.7	<0.0001 **
Aldosterone (pmol/L)	335.0 ± 182.0	1142.0 ± 615.0	<0.0001 **
Follow-up (months): 11.0 ± 1.7, Min: 9, Max: 14			
Eplerenone (mg/day):112.0 ± 59.0, Min: 50 mg, Max: 250 mg			

SBP: Systolic blood pressure; DBP: diastolic blood pressure: Min: minimum, Max: maximum, ** highly significant “*p*” value.

## Data Availability

Data available on request due to restrictions (privacy of personal data).
